# For Stage II Node-Positive Breast Cancer, is it Worthwhile to Consider Adjuvant Radiotherapy Following Mastectomy?

**DOI:** 10.3389/fonc.2014.00326

**Published:** 2014-11-19

**Authors:** Mohammed A. M. Osman, Mohammad S. Elkady, Khalid E. Nasr

**Affiliations:** ^1^General Organization For Teaching Hospitals and Institutes, Cairo, Egypt; ^2^Department of Oncology, Ain Shams University, Cairo, Egypt

**Keywords:** radiotherapy, breast, survival, group, toxicity

## Abstract

**Purpose:** To evaluate overall survival (OS), progression-free survival (PFS), loco-regional recurrence (LRR), and toxicities for early breast-cancer patients with one to three positive axillary lymph nodes, by the addition of radiotherapy to adjuvant chemotherapy.

**Patients and methods:** Patients were eligible for enrollment into the study if they had pathologically proven stages II breast cancer, with one to three positive axillary lymph nodes. Patients were assigned to one of the two groups; Group 1; adjuvant chemotherapy then radiotherapy, and group 2; adjuvant chemotherapy only.

**Results:** Between September 2008 and August 2014, 75 patients were enrolled. Forty patients group 1, and 35 group 2. The 4-year OS for group 1, and two were 77.5 and 71.4%, respectively. The 4-year PFS for group 1 and 2 were 72.5 and 60%, respectively. During the 54 months follow-up period, 11 patients from group 1 had recurrence (three locoregional, seven metastatic, and one both), and 14 patients from group 2 had recurrence (seven locoregional, three metastatic, and four both). The distant metastasis rate was the same in the two groups. However, the metastasis sites were different in the two groups.

**Conclusion:** The addition of radiotherapy in stage II breast cancer with one to three positive lymph nodes improved the PFS, and LRR. Radiotherapy improved OS in patients with high-risk features.

## Introduction

Breast cancer is the most frequent malignant tumor in women worldwide. In Egypt, it is the most common cancer among women, representing 18.9% of total cancer cases. More than 50% of patients present as early stages, thanks to advances that had recently made in the strategies of screening and early detection. A study by Salem et al. (1), showed that, in Egypt, 11% of patients presented as stage I, 30% stage II, 33% stage III, and 26% stage IV (1, 2).

Radiation therapy (RT) is frequently recommended for patients with early breast cancer who had undergone mastectomy. Guidelines from the American Society of Clinical Oncology (ASCO), the National Comprehensive Cancer Network (NCCN), and the American College of Radiology (ACR), all recommend for post-mastectomy RT for patients with four positive lymph nodes. However, these patients account for only a minority of node-positive disease. Unfortunately, all the three guidelines reported that insufficient evidence still exist to make any evidence-based post-mastectomy recommendations for patients with one to three positive nodes (2–5).

Considering RT in early breast cancer with one to three positive axillary lymph nodes remains unclear. Till recently, studies were unable to provide definite answers regarding this controversial issue (6–8).

### Study objectives

To evaluate the overall survival (OS), progression-free survival (PFS), loco-regional recurrence (LRR), and toxicities for early breast-cancer patients with one to three positive axillary lymph nodes, who underwent mastectomy, and axillary lymph node dissection, followed by either adjuvant chemotherapy and radiotherapy, or only adjuvant chemotherapy.

The primary end point was survivals benefits (PFS and OS). The secondary end points were LRR and toxicities.

## Materials and Methods

### Inclusion criteria

Female patients were eligible for enrollment into the study if they had pathologically proven breast cancer with one to three positive axillary lymph nodes on pathological examination. Status post mastectomy and axillary lymph node dissection. Patients should have stages II breast cancer (T1N1, T0N1, T2N1), with tumor size ≤5cm (pT1-2).

Further, for enrollment, patients should be between 17 and 65 years. Performance state had to be from 0 to 2 (ECOG). Patients should have adequate hematological, renal, and liver functions. Written informed consent from patients needed before proceeding in the trial. Female patients of childbearing potential must have a negative pregnancy test (serum β-HCG); and both the patient and her husband must employ effective contraceptive measures prior to the start of therapy until 4 weeks after the last dose of chemotherapy. Hormone receptor status has to be either ER or PR + ve, with HER 2 neu either positive or negative. Adjuvant chemotherapy should include anthracycline. Additionally, patients must have no concurrent malignancy.

Settings: The study was run in 2 educational institutes in Egypt; Ain Shams University Hospital, and Ismailia oncology teaching hospital.
Study design: Patients were assigned to one of the following groups;
Group 1: patients underwent adjuvant chemotherapy then radiotherapy,
Group 2: patients underwent adjuvant chemotherapy only.

### Radiotherapy

Radiotherapy was delivered by 3D conformal radiotherapy (CRT).

**Figure 1:**
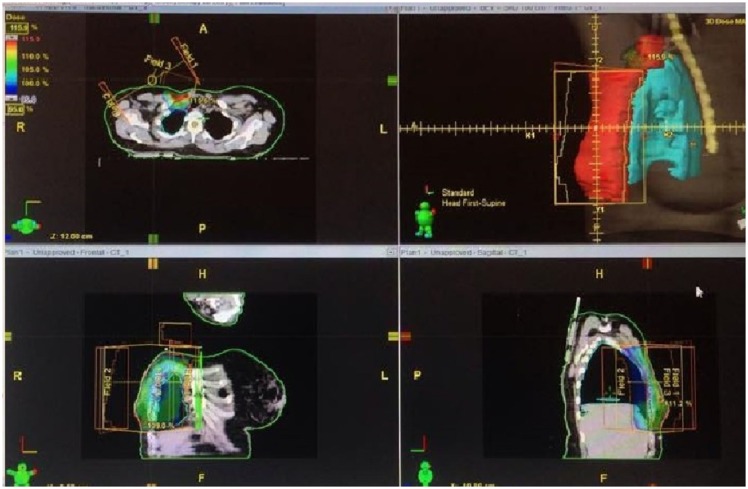
**CT planning for a patient in our study showing the field arrangements, doses**.

Positioning: The patient was placed supine with both arms above her head. Immobilization was maintained by the commercially available devices.
Simulation: Simulation was done using CT simulation technique.
Surgery scar, borders, and reference points were marked with radio-opaque wire before CT scanning. Clinical target volume (CTV), (OAR) definition as the following (Figure 1);
CTV was defined as the excision cavity plus 1.5 cm free resection margin.

The definitions of chest wall borders were guided by the following landmarks;

- Cranial: the caudal border of the clavicle head,
- Caudal: clinical reference, loss of CT apparent contralateral breast,
- Anterior: skin,
- Posterior: deep fascia,
- Lateral: clinical reference, mid axillary line,
- Medial: clinical reference.

The definitions of supraclavicular field borders are guided by the following landmarks;

- Cranial: inferior border of cricoid cartilage,
- Caudal: caudal edge of clavicular head/junction of brachiocephalic axillary veins,
- Anterior: sterocleidomastoid muscle,
- Posterior: anterior edge of the scalene muscle,
- Lateral: cranially: lateral edge of sterocleidomastoid muscle, and caudally: the first rib-clavicle junction,
- Medial: excludes thyroid and trachea.

Planning target volume (PTV) was equal the CTV plus 0.5–1 cm. The target was considered to be appropriately treated if the PTV was enclosed within the 95–105% isodose line.

### Organs at risk

The organs at risk (OAR) included the heart major blood vessels, ipsilateral, contolateral lungs, contralateral breast, cervical cord, and brachial plexus. Radiotherapy planning had to be adjusted to avoid OAR (9).

#### Field arrangement

Radiotherapy was delivered to the chest wall, and the supraclavicular fields using the same isocenter (mono-isocentric technique). Chest wall irradiation was done using two tangential fields, with the use of wedges as indicated. Multi-leaf collimator applied for optimally adjusting the therapeutic field to the tumor area. Supraclavicular Field irradiation was done by an anterior oblique field angled slightly away from the spinal cord. A humeral head block was added to block the humeral head and acromioclavicular joint.

#### Dose

The intended dose was 50 Gy, given in 25 fractions over a period of 5 weeks by conventional fractionation (2Gy/fraction).

RT machine was Linear accelerator 6-9 MV (10, 11).

After RT, all the patients were put on hormonal therapy. Additionally, those who had HER2 + ve disease continued on trastuzumab for 1 year.

### Follow-up

After finish of the treatment protocol, patients were followed-up according to the NCCN recommendations; by regular clinic visits every 4–6 months for the first 5 years, then annually thereafter. In each visit, patients were evaluated by history, physical examination. Annual X ray mammography, annual gynecological assessment for those on tamoxifin, and frequent DEXA scanning for those on aromatase inhibitors (5).

### Toxicity

Toxic effects were graded according to the National Cancer Institute Common Toxicity Criteria, version 2.0. Early toxicities were defined as toxicities that occurred during treatment till 8 weeks post radiotherapy. Late toxicities referred to those occurred >8 weeks after finish of treatment protocol (12).

### Statistical analysis

All calculations were carried out using prism-6 software for windows. All analyses were carried by intention to treat. Mean and median values were used for the description of continuous data. For comparison between the two group characters, *t*-test, and *p*-value were used. OS and PFS for each arm were analyzed by the Kaplan–Meier method. Further, they were compared using the log rank and Wilcoxon tests.

The univariate cox hazard analysis was applied to evaluate the effect of pre-specified prognostic factors including age, performance state, histopathological type, grade, stage, HER status, numbers of positive lymph nodes, and invasion on OS, PFS, recurrence, and deaths.

*p*-Value was significant at ≤0.05. Further to that, the study power was checked using SPSS-19 software.

Overall survival was measured from the time of randomization till death or the last follow-up visit. PFS was measured from the time of randomization till relapse, recurrence, or the last follow-up visit. LRR was defined as reappearance of local or regional tumor (in the chest wall, axilla, supraclavicular, or infraclavicular area) without distant metastases.

## Results

Between September 2008 and August 2014, 75 patients were enrolled in the current study. Forty patients were assigned to treatment group 1, and 35 patients were assigned to group 2. All the 75 patients fulfilled our eligibility criteria. The mean age was 52.4 years (range 29–65 years). The median performance status was 0 (range 0–2). All the 75 patients had stage II (4 T0N1, 29 T1N1, 42 T2N1) (5%, 39 and 56%, respectively) (Table 1).

**Table 1:** **Patients and diseases characteristics of each treatment group**.

Characteristics	Group 1		Group 2		*P*-value
	Number	%	Number	%	
**Age**
17– <40	1	2.5%	0	0%	–
40–49	12	30%	11	31%	0.2
50–60	17	42.5%	14	40%	0.1
61–65	10	25%	10	29%	0.3
**Mean age**	52	–	53	–	
**Performance status (ecog)**
0	20	50%	17	48.5%	0.2
1	15	37.5%	14	40%	0.1
2	5	12.5%	4	11.5%	0.1
**Performance status (median)**	0	–	0	–	
**Pathological classification**
Ductal	29	72.5%	26	74%	0.09
Lobular	11	27.5%	9	26%	0.06
Other	0		0		
**Pathological grade**
1	5	12.5%	6	17%	0.1
2	15	37.5%	19	54%	0.09
3	20	50%	10	28.5%	0.03
**Grade mean, median**	2.3, 3	–	2, 2	–	
**Tumor stage**
pT0N1	2	5%	2	6%	0.2
pT1N1	10	25%	19	54%	0.05
pT2N1	28	70%	14	40%	0.01
**Tumor stage median**	pT2N1		pT1N1		
**Lymph node number**
1	4	10%	7	20%	0.2
2	16	40%	15	42%	0.2
3	20	50%	13	37%	0.06
**Lymph node number median**	3		2		
**Her 2 neu**
Positive	2	5%	2	6%	0.1
Negative	38	95%	33	94%	0.09
**Invasion**
Lymphatic	15	37.5%	12	34%	0.1
Vascular/Peri-nural	10	25%	9	25%	0.2

### Treatment protocol

All the 75 patients underwent modified radical mastectomy, with axillary lymph node dissection (level I, II). Axillary lymph node dissection was done after sentinel lymph node examination in 50 patients by frozen section during the operation. A median of 10 lymph nodes were removed (range 5–16).

Following mastectomy, all our patients underwent adjuvant chemotherapy at mean time of 4 weeks. Chemotherapy regimes used included FAC (12 patients of group 1 and 13 patients group 2), AC (6 patients of group 1 and 2 patients group 2), and sequential AC, taxene (20 patients of group 1 and 20 patients group 2).

Additionally, two patients of group 1 received adjuvant CMF after the first cycle of FAC. The first patient developed prolonged febrile neutropenia grade IV, prolonged grade IV vomiting after cycle 1, and the second patient, who had history of rheumatic mitral regurgitation, developed low ejection fraction (EF) after cycle 1.

Further, 2 patients received adjuvant trastuzumab for HER-2 neu positive status (one group 1, one group 2).

### Radiotherapy

Radiotherapy was initiated at mean time of 2 weeks (range 2–3 weeks) after the last chemotherapy cycle for group 1 patients. Radiotherapy was given to all patients by 3D CRT, with CT simulation. The median RT dose was 50 Gy, in 25 fractions for all patients of group 1 (2Gy/fraction) (range 45–50 Gy).

Following adjuvant treatment, the two patients with HER + ve disease continued the full year of adjuvant Trastuzumab. Adjuvant hormonal therapy was given to our patients in the form of tamoxifin (25 patients) and sequential tamoxifin, armatase inhibitors (50 patients).

### Survival data

The median follow-up period was 54 (range 50–60 months).

**Figure 2:**
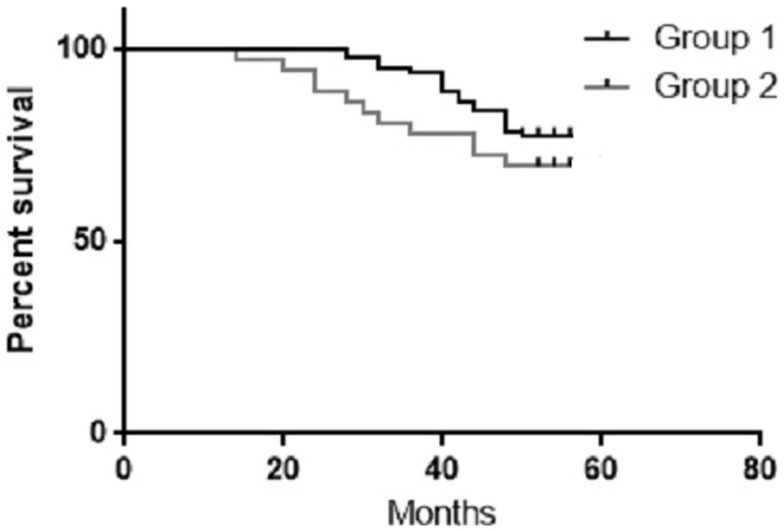
**The 4-year OS for the study groups – *p*-value: 0.2, chi square 1.29**.

The 4-year OS for group 1 and 2 were 77.5 and 71.4%, respectively (Figure 2). During the follow-up period, nine patients died from group 1 (8 cancer recurrence, and 1 cardiovascular cause), and 10 patients from group 2 died (9 cancer recurrence and 1 pulmonary embolism).

**Figure 3:**
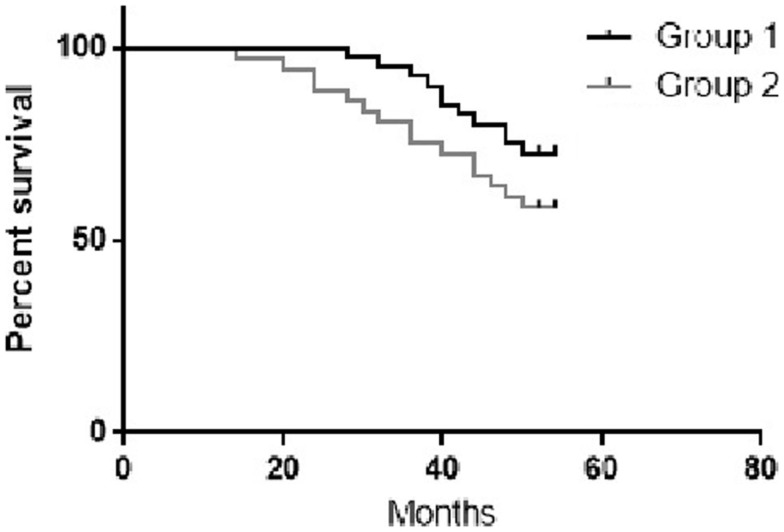
**The 4-year PFS for the study groups – *p*-value 0.03, chi square 4.28**.

The 4-year PFS for group 1 and 2 were 72.5 and 60% respectively (Figure 3).

For subgroup analysis, Tables 2 and 3 showed the 4-year OS, 4-year PFS categorized by the prognostic features.

**Table 2:** **The 4-year OS for each treatment group categorized by the prognostic factors**.

	Group 1%	Group 2 %	*P*-value
**Age**			
17– <40	100	–	–
40–49	75	72	0.07
50–60	76	78	0.1
61–65	70	60	0.01
**Performance status**			
0	90	88	0.3
1	73	64	0.03
2	40	25	0.01
**Pathological classification**			
Ductal	82	76	0.04
Lobular	63	55	0.04
**Pathological grade**			
1	100	83	0.03
2	73	78	0.3
3	75	50	0.009
**Tumor stage**			
pT0N1	100	50	0.001
pT1N1	80	73	0.06
pT2N1	75	71	0.2
**Lymph node number**			
1	75.5	71	0.2
2	87	78	0.04
3	70	64	0.03
**Her 2 neu**			
Positive	50	50	0.3
Negative	78	72	0.09
**Invasion**			
Lymphatic	67	50	0.02
Vascular/perinural	50	55	0.4

**Table 3:** **The 4 year PFS for each treatment group categorized by the prognostic factors**.

	Group 1%	Group 2 %	*P*-value
**Age**			
17– <40	100	–	–
40–49	75	73	0.3
50–60	70	64	0.03
61–65	60	40	0.008
**Performance status**			
0	85	76	0.05
1	67	57	0.007
2	40	0	0.001
**Pathological classification**			
Ductal	75	69	0.05
Lobular	63	33	0.001
**Pathological grade**			
1	80	67	0.02
2	73	68	0.06
3	70	40	0.001
**Tumor stage**			
pT0N1	100	50	0.001
pT1N1	70	63	0.06
pT2N1	75	57	0.003
**Lymph node number**			
1	75	57	0.03
2	75	67	0.02
3	70	53	0.003
**Her 2 neu**			
Positive	0	0	0.1
Negative	76	63	0.03
**Invasion**			
Lymphatic	67	25	0.009
Vascular/perinura	60	55	0.2

During the 54 months follow-up period, 11 patients from group 1 developed breast-cancer recurrence (3 locoregional, 7 metastatic, and 1 both), and 14 patients from group 2 had recurrence (7 locoregional, 3 metastatic, and 4 both) (Tables 4 and 5).

**Table 4:** **The relative risk of death, and recurrence (all causes) grouped by risk factors**.

	Death relative risk		Recurrence relative risk	
	Relative risk	*P*-value	Relative risk	*P*-value
**Age**			
<60	1.3	0.05	1.2	0.08
>60	1.1	0.1	1.1	0.1
**Performance status**			
<2	1	0.2	1.14	0.1
2	1.6	0.02	2	0.001
**Pathological classification**			
Ductal	1	0.2	1	0.2
Lobular	1.1	0.09	1.9	0.01
**Pathological grade**			
1,2	1	0.3	1.1	0.1
3	1.5	0.05	1.7	0.03
**5-Tumor stage**			
pT0N1	1.9	0.001	1.9	0.001
pT1N1	1	0.2	1.1	0.1
pT2N1	1	0.09	1.3	0.03
**Lymph node number**			
1	1	0.09	1.2	0.05
2, 3	1.2	0.05	1.3	0.04
**Her 2 neu**			
Positive	1	0.4	1	0.04
Negative	1.1	0.3	1.25	0.04
**Invasion**			
Lymphatic	1.34	0.02	2	0.001
Vascular/perinural	0.9	0.3	1.1	0.2

**Table 5:** **Characters of patients who developed locoregional recurrence during our follow up period for each treatment group**.

	Group 1			Group 2						
	1	2	3	1	2	3	4	5	6	7
**Age**										
17– <40										
40–49						X				
50–60			X	X	X		X			
61–65	X	X						X	X	X
**Performance status**										
0						X	X			
1	X	X		X	X			X		
2			X						X	X
**Pathological classification**										
Ductal	X	X		X	X				X	X
Lobular			X			X	X	X		
**Pathological grade**										
1										
2	X						X	X		X
3		X	X	X	X	X			X	
**Tumor stage**										
pT0N1										
pT1N1		X		X		X	X		X	
pT2N1	X		X		X			X		X
**Lymph node number**										
1										
2			X		X		X	X		
3	X	X		X		X			X	X
**Her 2 neu**										
Positive	X	X			X		X			
Negative			X	X		X		X	X	X
**Invasion**										
Lymphatic	X		X	X		X	X	X		X
Vascular/perinural	X				X					X

The distant metastasis rate was the same in the two groups (20% in both). However, the metastasis sites were different in the two groups. The sites of distant metastasis for group 1 were bone, liver, and lung, respectively (55, 30, and 15%, respectively). The sites of distant metastasis for group 2 were liver, lung, and bone, respectively (60, 25, and 15%, respectively).

### Toxicities

#### Early toxicity

##### Radiotherapy side effects

Radiotherapy delay happened in four patients (10%). The mean delay time was 1 week (range 5–14 days). No patient discontinued from RT for side effect. No deaths occurred related to treatment (Table 7).

#### Late toxicities

##### For group 1

During the 54 months follow-up period, four patients from group 1 developed lymphedema grade I–II (10%). No patient developed lung toxicity or brachial plexopathy. One patient from group 1 died from congestive cardiac failure at 50 months, to note that patient was known to have long history of rheumatic mitral regurgitation, and her EF was 45% before any treatment. EF dropped to 40% after the first cycle of FAC chemotherapy. EF was maintained at 40% after radiotherapy. Three years later, her EF was maintained at 40%. At 48 months, her EF dropped to 30%.

##### For group 2

During the same follow-up period, two patients from group 2 had lymphedema grade I (5.7%). One of them developed cellulitis on top of lymphedema. No other events were noticed to suggest delayed toxicities (Tables 6 and 7).

**Table 6:** **Grade 3, 4 early toxicities for group 1, 2**.

Side effect	Group 1		Group 2	
	Grade 3 (%)	Grade 4 (%)	Grade 3 (%)	Grade 4 (%)
Leukoneutropenia	10	0	11	0
Anemia	2.5	0	0	0
Thrombocytopenia	2.5	0	3	0
Febrile neutropenia	5	0	3	0
Nausea, Vomiting, GIT Upset	17.5	2.5	14	0
Mucositis	15	0	11	0
Dermatitis	7.5	0	0	0
Other (discomfort, swelling)	2.5	0	0	0

**Table 7:** **Grade 3, four early radiotherapy related side effects and their percentage in group 1**.

Side effect	Group 1	
	Grade 3 (%)	Grade 4 (%)
Leukoneutropenia	5	0
Thrombocytopenia	0	0
Febrile neutropenia	2.5	0
Nausea, vomiting, GIT upset	10	0
Mucositis	7.5	0
Dermatitis	7.5	0
Other (discomfort, swelling)	2.5	0

## Discussion

Radiotherapy in early breast cancer has been debatable for decades. While studies had showed its role in improving loco-regional control, its impact on OS is still unclear (13, 14).

The promising results of RT was supported by the results of studies of Cosar et al. (15) and Ragaz et al. (8), who reported that, addition of RT to chemotherapy for one to three node-positive breast cancer, improved the 5-year loco-regional control, and PFS. Further, the former study showed improvement of OS by 15% at 5 years, which was found to be non-statistically significant (*p* = 0.087), and the later showed improvement by 6% at 5 years, that was as well non-statistically significant (*p* = 0.1) (8, 15).

Beside these individual studies, number of systemic reviews and metaanalyses also demonstrate an absolute survival benefit of approximately 5–10% with the addition of RT (9, 14, 16, 17).

A recent metanalaysis conducted by the Early Breast-Cancer Trialists’ Collaborative Group (EBCTCG) in women who had one to three positive nodes. They reported that post-mastectomy RT reduced the recurrence rate by 32% and reduced the breast-cancer mortality rate by 20% (18).

On the other hand, an important meta-analysis by EBCTCG (19), that included 2000 women with early breast cancer, failed to show any improvement in OS after 20 years of follow up (19).

Many reasons can explain why the improvement in PFS was not reflected on definite improvement in OS. Cuzik et al. (20), reported that, there were late excess cardiac deaths that mask the potential reduction in deaths from breast cancers. However, excess cardiac deaths did not appear to be occurring in recent trials that used modern planning techniques including CRT. This might explain why the recent metaanalysis of EBCTCG (18), showed clear improvement in survival (19–21).

Another clinical evidence to confirm the above point that RT does not really increase late cardiac deaths, came from the metaanalysis of Van de Steene et al. (14), in which they reanalyzed the data of the EBCTCG (19) to include only recent trials, and they showed substantial reduction in the risk of mortality associated with radiotherapy by 12.4% (*p* < 0.001) (14).

Additional supporting evidence of the benefits of RT on OS came from the hypothesis that, if the burden of distant metastasis can be reduced by systemic therapy, then RT given to the loco-regional sites might prevent secondary dissemination (20, 21).

Finally, another evidence to support the benefit of RT on OS came from the role of RT after breast conservative surgery. A metaanalysis of Whelan et al. (22), showed that the addition of RT to systemic therapy statistically significantly reduced the risk of mortality after breast conservative surgery (odds ratio 0.83; *p* = 0.04). These results had led to the general acceptance that RT given after breast conservative surgery had favorable effects on survival (22).

Our study included Egyptian patients with early breast cancer from two educational institutes in Egypt, and patients were categorized into two groups. There was no statistical difference between the two groups in relation to their distribution to the pre-determined risk factors. Further, there were no ethnic or geographical differences among the two groups. The statistical power of the study was 0.72. Although the sample size was relatively small, the power of the current trial was relatively high. This was attributed to the high reliability, as well as the control of the confounding variables among the two groups. One of the reasons for the relatively low sample size that, in Egypt our breast-cancer screening program, that is supposed to detect cancer in early stages, is not yet well established or generalized, with only pilot trial launched in Ismailia oncology teaching hospital in 2012/2013.

Our inclusion criteria was strict to include only patients with one to three lymph node-positive stage II breast cancer, to avoid any other risk factors that may flaw the results.

In our study, we avoided giving RT by hypofractionation as this approach was not standardized at time of randomization. Further, we avoided giving radiotherapy by higher dose per fraction that may be associated with more toxicity profile especially late ones.

Our results indicated that, the addition of postoperative RT to systemic therapy after mastectomy for stage II breast-cancer significantly reduced LRR by 12.5%, and improved the PFS by 12.5% at 4 years.

For our OS results, RT improved the OS at 4 years by 6.1%, and that was found to be non-statistically significant. On the subgroup analysis, RT improved OS in the high-risk group of patients who had performance state 2, pathological grade 3, two to three positive lymph nodes, and those with lymphatic invasion. This was found statistically significant by univariate analysis.

The subgroup analysis for Tables 2 and 3 were done using univariate analysis. Mulitvariate analysis was not feasible because of the relatively small sample size.

Our study showed that upon recurrence, the distant metastasis rate was the same in the two groups (20% in both). However, the sites of metastasis were different between the two groups (Bone group 1 and liver group 2). The significance for that, as well as the correlation of that with RT is not yet clear.

Regarding the toxicity profile, our study confirmed that, RT by conformal 3D radiotherapy was generally tolerated well, and associated with mild toxicity profile.

## Conclusion

The addition of radiotherapy to adjuvant chemotherapy in stage II breast cancer with one to three positive lymph nodes improved the PFS, and LRR. Radiotherapy improved OS only in patients with high-risk features including those with PS 2, two to three positive axillary lymph nodes, and those with lymphatic invasion.

## Conflict of Interest Statement

The authors declare that the research was conducted in the absence of any commercial or financial relationships that could be construed as a potential conflict of interest.

## References

[1] Salem A Salem MA Abbass H Breast cancer: surgery at the south Egypt cancer institute. Cancers (2010) 2:1771–8 10.3390/cancers2041771 24281200 PMC3840361

[2] Overgaard M Jensen MB Overgaard J Hansen P Rose C Andersson M Postoperative radiotherapy in high-risk postmenopausal breast-cancer patients given adjuvant tamoxifen: Danish breast cancer cooperative group DBCG 82c trial. Lancet (1999) 353(9165):1641–8 10.1016/S0140-6736(98)09201-0 10335782

[3] Ragaz J Jackson SM Le N Plenderleith IH Spinelli JJ Basco VE Adjuvant radiotherapy and chemotherapy in node-positive premenopausal women with breast cancer. N Engl J Med (1997) 337(14):956–62 10.1056/NEJM199710023371402 9309100

[4] Recht A Edge SB Solin LJ Robinson DS Estabrook A Fine RE Postmastectomy radiotherapy: clinical practice guidelines of the American society of clinical oncology. J Clin Oncol (2001) 19(5):1539–69. 11230499 10.1200/JCO.2001.19.5.1539

[5] NCCN Clinical Practice Guidelines in Oncology, Breast Cancer, Version 3. (2014). Available from: http://www.nccn.org/professionals/physician_gls/pdf/breast.pdf

[6] Taylor ME Haffty BG Rabinovitch R Arthur DW Halberg FE Strom EA ACR appropriateness criteria on postmastectomy radiotherapy expert panel on radiation oncology-breast. Int J Radiat Oncol Biol Phys (2009) 73(4):997–1002. 10.1016/j.ijrobp.2008.10.080 19251087

[7] Overgaard M Nielsen HM Overgaard J Is the benefit of postmastectomy irradiation limited to patients with four or more positive nodes, as recommended in international consensus reports? A subgroup analysis of the DBCG 82 b&c randomized trials. Radiother Oncol (2007) 82(3):247–53 10.1016/j.radonc.2007.02.001 17306393

[8] Ragaz J Olivotto IA Spinelli JJ Phillips N Jackson SM Wilson KS Locoregional radiation therapy in patients with high-risk breast cancer receiving adjuvant chemotherapy: 20-year results of the British Columbia randomized trial. J Natl Cancer Inst (2005) 97(2):116–26 10.1093/jnci/dji217 15657341

[9] Martin D Dordai D Tănăsescu R Popa C Grecea D Bogdanet V CT simulation for radiotherapy in breast cancer patients. J Radiother Med Oncol (2011) 17(1):13–22.

[10] Smith BD Douglas W Arthur DW Buchholz TA Haffty BG Hahn CA Accelerated partial breast irradiation consensus statement from the American society for radiation oncology (ASTRO). Int J Radiat Oncol Biol Phys (2009) 74(4):987–1001. 10.1016/j.ijrobp.2009.02.031 19545784

[11] Polgar C Van Limbergen E Potter R Kovács G Polo A Lyczek J Patient selection for accelerated partial-breast irradiation (APBI) after breast-conserving surgery: recommendations of the groupe Européen de curiethérapie-European society for therapeutic radiology and oncology (GEC-ESTRO) breast cancer working group based on clinical evidence. Radiother Oncol (2010) 94(3):264–73 10.1016/j.radonc.2010.01.014 20181402

[12] Common Toxicity Criteria (CTC) Version 2.0. (1999). Available from: http://www.eortc.be/services/doc/ctc/ctcv20_4-30-992.pdf

[13] Gebski V Lagleva M Keech A Simes J Langlands AO Survival effects of postmastectomy adjuvant radiation therapy using biologically equivalent doses: a clinical perspective. J Natl Cancer Inst (2006) 98:26–38 10.1093/jnci/djj002 16391369

[14] Van de Steene J Soete G Storme G. Adjuvant radiotherapy for breast cancer significantly improves overall survival: the missing link. Radiother Oncol (2000) 55:263–72. 10.1016/S0167-8140(00)00204-8 10869741

[15] Cosar R Uzal C Tokatli F Denizli B Saynak M Turan N Postmastectomy irradiation in breast in breast cancer patients with T1-2 and 1-3 positive axillary lymph nodes: is there a role for radiation therapy. Radiat Oncol (2011) 6:28–35. 10.1186/1748-717X-6-28 21450076 PMC3072917

[16] Rutqvist LE Rose C Cavallin-Stahl E. A systematic overview of radiation therapy effects in breast cancer. Acta Oncol (2003) 42:532–45. 10.1080/02841860310014444 14596511

[17] Darby S On behalf of the Early Breast Cancer Trialists’ Collaborative Group Overview of the randomised trials of radiotherapy in early breast cancer (Abstract). Presented at the 32nd Annual San Antonio Breast Cancer Symposium San Antonio, TX (2009).

[18] McGale P Taylor C Correa C On behalf of the Early Breast Cancer Trialists’ Collaborative Group. Effect of radiotherapy after mastectomy and axillary surgery on 10-year recurrence and 20-year breast cancer mortality: meta-analysis of individual patient data for 8135 women in 22 randomised trials. Lancet (2014) 383(9935):2127–35. 10.1016/S0140-6736(14)60488-8 24656685 PMC5015598

[19] EBCTCG. Favourable and unfavourable effects on long term survival of radiotherapy for early breast cancer; an overview of the randomized trials. Lancet (2000) 355:757–70. 10832826

[20] Cuzik J Stewart H Rutqvist L Houghton J Edwards R Redmond C Cause specific mortality in long term survivors of breast cancer who participated in trials of radiotherapy. J Clin Oncol (1994) 12:447–53. 8120544 10.1200/JCO.1994.12.3.447

[21] Krauss DJ Kestin LL Raff G Yan D Wong J Gentry R MRI-based volumetric assessment of cardiac anatomy and dose reduction via active breathing control during irradiation for left-sided breast cancer. Int J Radiat Oncol Biol Phys (2005) 61:1243–50. 10.1016/j.ijrobp.2004.10.012 15752906

[22] Whelan TJ Julian J Wright J. Does radiation therapy improve survival in breast cancer? A Meta-analysis. J Clin Oncol (2000) 18:1220–9. 10715291 10.1200/JCO.2000.18.6.1220

